# Group Testing with Blocks of Positives and Inhibitors

**DOI:** 10.3390/e24111562

**Published:** 2022-10-30

**Authors:** Thach V. Bui, Isao Echizen, Minoru Kuribayashi, Tetsuya Kojima, Thuc D. Nguyen

**Affiliations:** 1 Department of Computer Science, National University of Singapore, Singapore 117417, Singapore; 2 National Institute of Informatics, Tokyo 101-8430, Japan; 3 Department of Information and Communication Engineering, University of Tokyo, Tokyo 113-8654, Japan; 4 Graduate School of Natural Science and Technology, Okayama University, Okayama 700-8530, Japan; 5 National Institute of Technology, Tokyo College, Hachioji, Tokyo 193-0997, Japan; 6 Faculty of Information Technology, University of Science, VNU-HCMC, Ho Chi Minh City 72711, Vietnam; 7 Faculty of Information Technology, Vietnam National University, Ho Chi Minh City 720300, Vietnam

**Keywords:** non-adaptive group testing, inhibitors, combinatorics, sub-linear algorithms, sparse recovery

## Abstract

The main goal of group testing is to identify a small number of specific items among a large population of items. In this paper, we consider specific items as positives and inhibitors and non-specific items as negatives. In particular, we consider a novel model called *group testing with blocks of positives and inhibitors*. A test on a subset of items is positive if the subset contains at least one positive and does not contain any inhibitors, and it is negative otherwise. In this model, the input items are linearly ordered, and the positives and inhibitors are subsets of small blocks (at unknown locations) of consecutive items over that order. We also consider two specific instantiations of this model. The first instantiation is that model that contains a single block of consecutive items consisting of exactly known numbers of positives and inhibitors. The second instantiation is the model that contains a single block of consecutive items containing known numbers of positives and inhibitors. Our contribution is to propose efficient encoding and decoding schemes such that the numbers of tests used to identify only positives or both positives and inhibitors are less than the ones in the state-of-the-art schemes. Moreover, the decoding times mostly scale to the numbers of tests that are significantly smaller than the state-of-the-art ones, which scale to both the number of tests and the number of items.

## 1. Introduction

Group testing [1] was first introduced to reduce time and cost of testing draftees who were possibly positive for syphilis. In this problem, the number of syphilitic draftees is outnumbered by the number of non-syphilitic draftees. The main idea of group testing is instead of testing draftees individually, sets of draftees are pooled and tested. If the test outcome of a pool is positive, then there exists at least one draftee in that pool that is syphilitic and none of the draftees in the pool are syphilitic otherwise. Since this seminal work, group testing has been usually treated as a problem of identifying a small number of specific items in a large population of items. The specific items depend on context and affect how a test on a subset of items is positive or negative.

There are two general strategies for designing tests [2]. The first is adaptive group testing in which the design of a test depends on the designs of the previous tests. This approach usually attains an information-theoretic bound for the number of tests but consumes a substantial amount of time for implementation because of several design stages. To remedy its time-consuming nature while achieving a relatively low number of tests, non-adaptive group testing (NAGT) is used. In this strategy, all tests are designed independently and can be performed in parallel. Because of its advantage, NAGT has been used in a wide range of applications, such as computational and molecular biology [2,3], networking [4], COVID-19 [5,6], and neuroscience [7]. In this work, our focus is on the non-adaptive testing strategy.

NAGT can be represented by a t×n binary matrix $T=(t_{ij})$, where *n* is the number of items and *t* is the number of tests. An entry $t_{ij}=1$ means that item (column) *j* belongs to test (row) *i*, and $t_{ij}=0$ means otherwise. The *j*th item is represented by the *j*th column of the matrix. The procedure to produce the measurement matrix is called *construction*, the procedure to obtain the outcomes of tests using the measurement matrix is called *encoding*, and the procedure to recover specific items from the outcomes is called *decoding*. A measurement matrix is *random* if some tests are generated by a probabilistic scheme, whereas it is *deterministic* if every test is deterministic. A measurement matrix is *strongly explicit* (explicit) if it takes the time and space polynomial of the number rows (respectively, the number rows and the number of columns) to generate a column in it.

Some distribution settings may apply on specific items. There are two common settings: (i) the probabilistic setting, in which there is some probability distribution used on specific items, and the identification error probability is allowed; and (ii) the combinatorial setting, which is our focus here, and no probability distribution is used on specific items.

Consider standard group testing in which specific items are only positives. Suppose a test on a subset of items is positive if the subset contains at least one positive and is negative otherwise. Throughout the paper, log refers to base 2 logarithms. If we give a population of *n* items up to *d* positives, then there are a number of works for attaining a low number of tests, say $t=O(d^{2}\log^{1+o(1)}n)$, and/or a fast decoding time, say poly(d,lnn) [8,9,10,11,12,13,14] in the combinatorial setting. In probabilistic settings, Bondorf [15] et al. show that the number of tests can be reduced to O(dlogn) with a decoding time of $O(d^{2}\log d\cdot \log n)$. Price and Scarlett [16] later improved the decoding time to O(dlogn).

### 1.1. New Model and Problem Definition

Because of the natural phenomenon in biology, a new type of item called *inhibitor* was introduced in group testing [3] and studied [17,18,19,20]. An inhibitor item causes a negative outcome for any test it is involved in. On the other hand, a test on a subset of items is positive if the subset does not contain any inhibitor and contains at least one positive.

Group testing with blocks of positives has been recently presented by Bui et al. [21], which is a generalization of group testing with consecutive positives [22,23,24,25,26,27]. In this model, input *n* items are linearly ordered, and all positives belong to at most *k* blocks of consecutive items and each block has up to *d* consecutive items.

**Figure 1:**
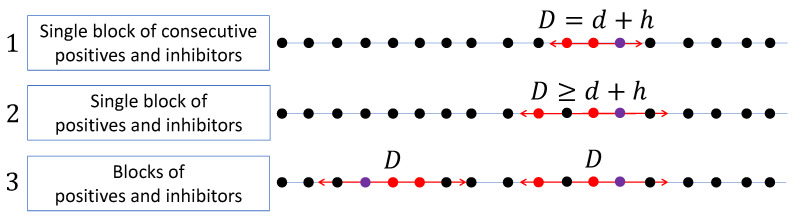
Three models for blocks of positives and inhibitors. Red, purple, and black dots represent positives, inhibitors, and negative items, respectively. A double arrow line stands for a block of *D* consecutive items. The first, second, and third models are a single block of consecutive positives and inhibitors, single block of positives and inhibitors, and blocks of positives and inhibitors. The second model is a generalization of the first model, and the third model is a generalization of the first two models.

Combining the two models above, we consider a novel model called *group testing with blocks of positives and inhibitors*. The input *n* items are linearly ordered. We sub-categorize the model into three models and illustrate them in Figure 1. The first model contains one block of d+h consecutive items and that block contains exactly *d* positives and *h* inhibitors. The second model, which is a general model of the first one, contains one block of D≥d+h consecutive items and that block contains up to *d* positives and *h* inhibitors. The third model, which is the most general one, contains multiple blocks, says *k*, of consecutive items in which each block of size up to D≥d+h contains up to *d* positives and *h* inhibitors. Note that the assumption on the known upper bounds for k,h, and *d* are obtained from previous statistics.

We formulate the three models above as follows. Sets of the form $C=\{c_{1},\ldots ,c_{k}\}$ used in this work are equipped with linear order $c_{i}≺c_{i+1}$ for 1≤i<k. We index the population of *n* items from 1 to *n*, namely N={1,2,…,n}. Let $x={\left(x_{1},\ldots ,x_{n}\right)}^{T}\in {\left\{-1,0,1\right\}}^{n}$ be the binary representation vector of *n* items, where $x_{j}=1$ indicates that item *j* is positive, $x_{j}=0$ indicates that item *j* is negative, and $x_{j}=-1$ indicates that item *j* is inhibitory. A test on a subset of items is positive if the subset contains at least one positive and does not contain any inhibitors. Otherwise, the test outcome is negative.

The test notation is denoted as ⊙. Let $p=\left(p_{1},\ldots ,p_{n}\right)\in {\left\{0,1\right\}}^{n}$ be the test representation vector. Then, the outcome vector of the test p with the input vector x, namely p⊙x, is positive (1) if there does not exist a *j* such that $p_{j}=1$ and $x_{j}=-1$, and there exists a $j^{\prime }$ such that $p_{j^{\prime }}=1$ and $x_{j^{\prime }}=1$. The test outcome is negative (0) otherwise. Given a measurement matrix M of size t×n and an input vector x, the corresponding outcome vector is $M⊙x={\left[y_{1},\ldots ,y_{n}\right]}^{T}$, where $y_{i}=M\left(i,:\right)⊙x$.

There are two common decoding types based on classification strategy. The first is to only identify the positives while the second is to identify both positives and inhibitors. Our objective is to find an efficient encoding and decoding scheme to satisfy two decoding types, i.e., minimizing the number of tests and the decoding time.

### 1.2. Contributions

Overview: We study group testing with blocks of positives and inhibitors and provide efficient encoding and decoding schemes to tackle it. By leveraging the knowledge of positives and inhibitors belonging to a small interval of size *D*, our objective is to identify the position of some positive, say $j^{*}$; then, one could claim that the indices of all positives and inhibitors must belong to the range from $\max\{1,j^{*}-D+1\}$ to $\min\{j^{*}+D-1,n\}$. To precisely identify positives and inhibitors, appropriate tests are designed to accomplish this task.

Our proposed scheme includes two procedures, which are the filtering and scrutinizing procedures. The tests in the filtering procedure remove most negative items and leave a subset(s) of size up to 2D that contains all positives, inhibitors, and probably some negatives. The tests in the scrutinizing procedure remove all negatives and then identify positives and inhibitors. The details of the two procedures are specified in accordance to each specific problem.

The contributions for a single block of (consecutive) positives and inhibitors is summarized in Theorem 1. The proofs for the results of the first and second model are described in Section 3 and Section 4.

**Theorem 1.**  *Let 1≤d,h,d+h≤n be integers. Suppose that a population of n linearly ordered items includes exactly d positives and h inhibitors in a block of D≥d+h items that are consecutive that order. When D=d+h (respectively, D≥d+h), there exists a deterministic and strongly explicit measurement matrix of size $O\left(h\log\frac{hn}{d+h}+d\right)\times n$ (respectively, O(Dlogn)×n) that can be used to identify all positives in $O\left(h\log\frac{hn}{d+h}+d\right)$ (respectively, O(Dlogn)) time. Moreover, it requires $O\left({\left(d+h\right)}^{3}\log\frac{n}{d+h}\right)$ (respectively, $O\left(D\log n+D^{3}\log\left(n/D\right)\right)$) tests to identify all positives and inhibitors in $O\left({\left(d+h\right)}^{4}\log\frac{n}{d+h}\right)$ (respectively, $O\left(D\log n+D^{4}\log\left(n/D\right)\right)$) time.*

The contribution for blocks of positives and inhibitors is summarized in the following theorem, which is proved later in Section 5.

**Theorem 2.**  *Let 1≤d,h,d+h≤D≤n be integers. Suppose that a population of n items is linearly ordered and the positives and inhibitors belong to blocks of consecutive items in which each block has a size of up to D and contains up to d positives and h inhibitors. Then, there exists a deterministic and strongly explicit measurement matrix of size $O(Dk^{2}\left(D+\log\frac{n}{D}\right)\log\frac{n}{D})\times n$ that can be used to identify all positives in $O(Dk^{2}\left(D+\log\frac{n}{D}\right)\log\frac{n}{D})$ time. Moreover, it requires $O(D^{2}k^{2}\left(D+\log\frac{n}{D}\right)\log\frac{n}{D})$ tests to identify all positives and inhibitors in time.*

$$O\left(Dk^{2}\log\frac{n}{kD}\left(D+\log\frac{n}{D}\right)+k^{4}D^{4}\log\frac{n}{kD}\right).$$

## 2. Preliminaries

Disjunct matrices were first introduced by Kautz and Singleton [28] as *superimposed codes* and then generalized by Stinson and Wei [29] and D’yachkov et al. [30]. We later use them for identifying both positives and inhibitors. Let the support set for vector $v=(v_{1},\ldots ,v_{w})$ be $\mathrm{supp}\left(v\right)=\left\{j|v_{j}\neq 0\right\}$ and $\left|v|=|\{j|v_{j}\neq 0\}\right|$. We denote M(i,:) and M(:,j) as the *i*th row and the *j*th column of matrix M. The formal definition of a disjunct matrix is as follows.

**Definition 1.**  *An m×n binary matrix M is called an (n,v,u)-disjunct matrix if, for any two disjoint subsets $S_{1},S_{2}\subset \left[n\right]$ such that $|S_{1}|=v$ and $|S_{2}|=u$, there exists at least one row in which there are all 1s among the columns in $S_{2}$ while all the columns in $S_{1}$ have 0s, i.e., $\left|{⋂}_{j\in S_{2}}\mathrm{supp}\left(M\left(:,j\right)\right)\backslash {⋃}_{j\in S_{1}}\mathrm{supp}\left(M\left(:,j\right)\right)\right|\geq 1$.*

Chen et al. [31] gave an upper bound on the number of rows for (n,v,u)-disjunct matrices as follows.

**Theorem 3**  ([31] Theorem 3.2)**.** *For any positive integers v,u, and n with x=v+u≤n, there exists a t×n(n,v,u)-disjunct matrix with the following.*

(1) $$\begin{array}{c} t\left(n,v,u\right)=O\left({\left(\frac{x}{u}\right)}^{u}{\left(\frac{x}{v}\right)}^{v}x\log\frac{n}{x}\right). \end{array}$$

Once u=1, (n,v,1)-disjunct matrices become *v*-disjunct matrices. The following theorem states the construction and decoding time for a *d*-disjunct matrix.

**Theorem 4**  ([11] Theorem 16)**.** *Let 1≤d≤n. Then, there exists a deterministic and explicit t×n d-disjunct matrix with $t=O(d^{2}\log n)$ that can be decoded in the polynomial time of t.*

## 3. Single Block of Consecutive Positives and Inhibitors

In this section, we consider the case when the positives and inhibitors are consecutive and the numbers of positives and inhibitors are known in advance. Set D=d+h.

### 3.1. Encoding Procedure

Set a=⌊D/(h+1)⌋ and κ=⌈n/a⌉. A super item, denoted as $\bar{\cdot }$, is a set of consecutive items. The *n* items are distributed into κ super items indexed from 1 to κ and each super item contains exactly *a* items, except that the last one may contain less than *a* items. Let $\bar{E}=\left\{\bar{1},\bar{2},\ldots ,\bar{\kappa }\right\}$ be the set of super items generated from N, where $\mathrm{set}\left(\bar{j}\right)=\left\{\left(j-1\right)a+1,\ldots ,ja\right\}$ for j=1,…,κ−1 and $\mathrm{set}\left(\bar{\kappa }\right)=\left\{\left(\kappa -1\right)a+1,\ldots ,n\right\}$. We then denote that $\chi_{\bar{E}}=\left(\chi_{1},\ldots ,\chi_{\kappa }\right)$ be the characteristic vector of $\bar{E}$, where $\chi_{j}=1$ if the test on $\bar{j}$ is positive and $\chi_{j}=0$ otherwise.

#### 3.1.1. Filtering Matrices

We create h+2 filtering matrices in the filtering procedure as follows. Let $F=[f_{1},\cdots ,f_{\kappa }]$ be an f×κ (indexing) binary matrix for which its *j*th column is the *f*-bit binary representation of integer *j*, where f=⌈log(κ+1)⌉. It is obvious that the index *j* is uniquely identified by $f_{j}$. We then generate h+2 binary matrices $F^{\left(u\right)}=\left[F_{1}^{\left(u\right)},\ldots ,F_{\kappa }^{\left(u\right)}\right]$ for u=1,…,h+2, such that column $F^{\left(u\right)}\left(:,j\right)$ is a zero vector if j≢umod(h+2) while $F^{\left(u\right)}\left(:,j\right)=f_{j}$ if j≡umod(h+2). For example, let n=12,d=4, and h=2. We obtain a=⌈(d+h)/(h+1)⌉=2 and κ=⌈n/a⌉=6. Since $F=[f_{1},f_{2},f_{3},f_{4},f_{5},f_{6}]$, we imply $F^{\left(1\right)}=\left[f_{1},0,0,0,f_{5},0\right]$, $F^{\left(2\right)}=\left[0,f_{2},0,0,0,f_{6}\right]$, $F^{\left(3\right)}=\left[0,0,f_{3},0,0,0\right]$, and $F^{\left(4\right)}=\left[0,0,0,f_{4},0,0\right]$. For every h+2 consecutive column in $F^{\left(u\right)}$, there exists only one non-zero column. Therefore, it is used to “isolate” each super item in the h+1 super items generated from the set of *D* positives and inhibitors.

Let $y_{F^{\left(u\right)}}={\left[y_{F^{\left(u\right)}}\left(1\right),\ldots ,y_{F^{\left(u\right)}}\left(f\right)\right]}^{T}$ be the outcome vector by using the testing matrix $F^{\left(u\right)}$ with the set of super items $\bar{E}$. In particular, if $F^{\left(u\right)}\left(i,j\right)=1$ (respectively, $F^{\left(u\right)}\left(i,j\right)=0$) then all items in the super item $\bar{j}$ (respectively, do not) belong to test *i*. Therefore, we obtain the following.

(2) $$y_{F^{\left(u\right)}}=F^{\left(u\right)}⊙\chi_{\bar{E}}.$$

#### 3.1.2. Sanitizing Matrices

In the sanitizing procedure, the measurement matrix depends on whether the objective is to identify positives only or to identify both positives and inhibitors. For the first objective, we design an s×n matrix S such that S(i,j)=1 if i≡jmods and S(i,j)=0 or, otherwise, where s=2D−1. In other words, each test contains items spaced 2D−1 apart in a linear order. For example, when n=12,d=4, and h=2, we obtain the following.

$$S=\left[\begin{array}{cccccccccccc} 1 & 0 & 0 & 0 & 0 & 0 & 0 & 0 & 0 & 0 & 0 & 1\\ 0 & 1 & 0 & 0 & 0 & 0 & 0 & 0 & 0 & 0 & 0 & 0\\ 0 & 0 & 1 & 0 & 0 & 0 & 0 & 0 & 0 & 0 & 0 & 0\\ 0 & 0 & 0 & 1 & 0 & 0 & 0 & 0 & 0 & 0 & 0 & 0\\ 0 & 0 & 0 & 0 & 1 & 0 & 0 & 0 & 0 & 0 & 0 & 0\\ 0 & 0 & 0 & 0 & 0 & 1 & 0 & 0 & 0 & 0 & 0 & 0\\ 0 & 0 & 0 & 0 & 0 & 0 & 1 & 0 & 0 & 0 & 0 & 0\\ 0 & 0 & 0 & 0 & 0 & 0 & 0 & 1 & 0 & 0 & 0 & 0\\ 0 & 0 & 0 & 0 & 0 & 0 & 0 & 0 & 1 & 0 & 0 & 0\\ 0 & 0 & 0 & 0 & 0 & 0 & 0 & 0 & 0 & 1 & 0 & 0\\ 0 & 0 & 0 & 0 & 0 & 0 & 0 & 0 & 0 & 0 & 1 & 0 \end{array}\right].$$

It is straightforward that every column in S (respectively, $F^{\left(u\right)}$) is deterministic and strongly explicit because each column in it can be generated in time and space of O(2D)=O(d+h) (respectively, O(logκ)).

For the second objective, i.e., the objective of identifying both positives and inhibitors, we design an additional matrix R along with matrix S. Let R be a r×n(n,2D−3,2)-disjunct matrix as defined in Definition 1. Therefore, we have $r=O\left(D^{3}\log\left(n/D\right)\right)=O\left({\left(d+h\right)}^{3}\log\left(n/\left(d+h\right)\right)\right)$ as in Theorem 3.

Let $y_{S}={\left[y_{S}\left(1\right),\ldots ,y_{S}\left(s\right)\right]}^{T}$ (respectively, $y_{R}={\left[y_{R}\left(1\right),\ldots ,y_{R}\left(r\right)\right]}^{T}$) be the outcome vector by using the testing matrix S (respectively, R) with input set *N*. In particular, we have the following.

(3) $$y_{S}=S⊙xandy_{R}=R⊙x.$$

### 3.2. Decoding Procedure and Correctness

We first approximately locate some positive items by using outcome vectors $y_{F^{\left(1\right)}},\ldots ,y_{F^{\left(h+1\right)}}$. Then, we can locate a set of up to 2D−1 items that contains all positives and inhibitors. We call this set *the set of interest*. By using $y_{S}$, we can exactly identify all positives in that set. Meanwhile, if $y_{R}$ is also used, all inhibitors are also identified. The details of the decoding procedure are as follows.

Let λ be an index such that $y_{F^{\left(\lambda \right)}}$ is not a zero vector. There always exists a λ. Indeed, because there are *h* inhibitors, *D* consecutive positives and inhibitors, and each super item contains up to ⌊D/(h+1)⌋ items, the total number of items contained in super items having inhibitors is up to h⌊D/(h+1)⌋<D. Therefore, there must exist a super item $\bar{\alpha }$ that does not contain any inhibitor but all positives for 1≤α≤κ. Let λ be the index such that $F^{\left(\lambda \right)}\left(:,\alpha \right)\neq 0$. Since two consecutive non-zero column in $F^{\left(u\right)}$ are space by h+2, $y_{F^{\left(\lambda \right)}}=F^{\left(\lambda \right)}⊙\chi_{\bar{E}}=F^{\left(\lambda \right)}\left(:,\alpha \right)$. Therefore, to identify α, we convert a non-zero vector $y_{F^{\left(\lambda \right)}}$ into a decimal number. The indices of all positives and inhibitors, thus, must belong to the range from $\max\{1,j^{*}-D+1\}$ to $\min\{j^{*}+D-1,n\}$. The decoding complexity of identifying λ is therefore O(hf).

Because of the construction of S, a matrix composed of 2D−1 consecutive columns in it is a permutation of a (2D−1)×(2D−1) identity matrix. Therefore, given the indices from max{1,α−D+1} to min{α+D−1,n}, one can identify which item is positive based on the corresponding outcome vector $y_{S}$. The decoding complexity of $y_{S}$ is, therefore, O(D)=O(d+h).

After identifying *d* positives, the set of interest contains up to 2D−1−d=d+2h−1 potential inhibitors. Because of the construction of R, for any two items and other 2D−3 items, there exists a test that contains the two items and does not contain the other 2D−3 items. Therefore, one could identify whether a potential inhibitor is truly an inhibitor by checking the row that contains it and whether it is a positive, in addition to checking that it does not contain the remaining items in the set of interest. Since there are up to 2D−1−d potential positives and the number of rows in R is *r*, this procedure to identify inhibitors takes O(r(2D−1−d))=O(r(d+h)).

### 3.3. Decoding Complexity and Number of Tests

As analyzed in the previous section, to identify the positives only, the number of required tests and the decoding complexity are as follows.

$$O\left((h+2)f+s\right)=O\left(h\log\frac{hn}{d+h}\right)+O\left(d+h\right)=O\left(h\log\frac{hn}{d+h}+d\right).$$

To identify both the positives and inhibitors, i.e., classify all items, the required number of tests is as follows.

$$\begin{array}{cc} (h+2)f+s+r & =O\left(h\log\frac{hn}{d+h}\right)+O\left(d+h\right)+O\left({\left(d+h\right)}^{3}\log\frac{n}{d+h}\right)\\ \; & =O\left({\left(d+h\right)}^{3}\log\frac{n}{d+h}\right). \end{array}$$

The corresponding decoding complexity is as follows.

$$O\left(hf\right)+O\left(d+h\right)+O\left(r\left(d+h\right)\right)=O\left({\left(d+h\right)}^{4}\log\frac{n}{d+h}\right).$$

## 4. Single Block of Positives and Inhibitors

In this section, we consider the case when the positives and inhibitors are not necessarily consecutive but belong to a small block (set) of consecutive items of size up to D≥d+h, where *d* and *h* are the maximum numbers of positives and inhibitors in the population of *n* items.

In the encoding procedure, we use the same techniques in Section 3.1 but adjust some parameters. In the filtering procedure, we set a=1, i.e., every super item reduces to an item. Therefore, κ is equal to *n*. Moreover, we create *D* filtering matrices, i.e., h+1 is replaced by *D*. In the sanitizing procedure, the parameter *s* in the s×n matrix S is set to be 2D−1. Matrix R is a r×n(n,2D−3,2)-disjunct matrix as defined in Definition 1. Therefore, we have $r=O(D^{3}\log\left(n/D\right))$ as in Theorem 3.

Since the decoding procedure and the proofs of correctness are as the same as in Section 3.2, we only pay attention for the required numbers of tests and the decoding complexities. Each matrix $F^{\left(u\right)}$ has a size of f×n, where f=⌈logn⌉, for u=1,…,D. The numbers of tests in matrices S and R are s=2D−1 and $r=O(D^{3}\log\left(n/D\right))$, respectively.

To identify the positives only, the number of required tests and the decoding complexity are as follows.

$$Df+s=O\left(D\log n\right)+O\left(D\right)=O\left(D\log n\right).$$

To identify both the positives and inhibitors, the required number of tests is described as follows.

$$\begin{array}{cc} Df+s+r & =O\left(D\log n\right)+O\left(D\right)+O\left(D^{3}\log\left(n/D\right)\right)\\ \; & =O\left(D\log n+D^{3}\log\left(n/D\right)\right). \end{array}$$

The corresponding decoding complexity is as follows.

$$O\left(Df\right)+O\left(d+h\right)+O\left(rD\right)=O\left(D\log n+D^{4}\log\left(n/D\right)\right).$$

## 5. Blocks of Positives and Inhibitors

In this section, we consider a model consisting of multiple blocks of positives and inhibitors, in which all positives and inhibitors belong to at most *k* special blocks of consecutive items and each block has up to *D* consecutive items.Moreover, each special block contains up to *d*positives and up to *h* inhibitors.

### 5.1. Encoding Procedure

We generate *D* sets from the set of *n* items N as follows. Set $N^{\left(u\right)}=\left\{u,D+u,2D+u,\ldots ,n_{u}\right\}$ and $x^{\left(u\right)}={\left(x_{u},x_{D+u},x_{2D+u},\ldots ,x_{n_{u}}\right)}^{T}$, where $n_{u}$ is the largest number smaller than *n* and $n_{u}\equiv u\;\mathrm{mod}\;D$, for u=1,…,D. It is obvious that $n_{u}=\left|N^{\left(u\right)}\right|\leq \left\lceil n/D\right\rceil$. Since each special block has up to *D* items and there are up to *k* special blocks, each set $N^{\left(u\right)}$ must contain up to *k* positives and inhibitors in total. Moreover, for each special block τ, there exists an index $u_{\tau }$ such that some positive item in that block belongs to $N^{\left(u_{\tau }\right)}$ because two consecutive items in $N^{\left(u\right)}$ are spaced apart by *D* and each special block has up to *D* items.

Let $M^{\left(u\right)}$ be an $m_{u}\times n_{u}$*k*-disjunct matrix. We then obtained $m_{u}=O\left(k^{2}\log n_{u}\right)$$=O(k^{2}\log\left(n/D\right))$ as in Theorem 4. Let $B^{\left(u\right)}$ be a $b\times n_{u}$ index matrix:

(4) $$B^{\left(u\right)}:=\left[\begin{array}{cccc} b_{1} & b_{2} & \ldots & b_{n_{u}}\\ {\bar{b}}_{1} & {\bar{b}}_{2} & \ldots & {\bar{b}}_{n_{u}} \end{array}\right]=\left[\begin{array}{ccc} B_{1}^{\left(u\right)} & \ldots & B_{n_{u}}^{\left(u\right)} \end{array}\right],$$

where $b=2\left\lceil \log n_{u}\right\rceil$, $b_{j}$ is the $\left\lceil \log n_{u}\right\rceil$-bit binary representation of integer j−1, ${\bar{b}}_{j}$ is the complement of $b_{j}$, and $B_{j}^{\left(u\right)}:=\left[\begin{array}{c} b_{j}\\ \bar{b_{j}} \end{array}\right]$ for $j=1,2,\ldots ,n_{u}$. Item *j* is characterized by column $B_{j}$ and that the weight of every column in B is $b/2=\left\lceil \log n_{u}\right\rceil .$ Furthermore, the index *j* is uniquely identified by $b_{j}$. For example, if we set $n_{u}=8$, $b=2\left\lceil \log n_{u}\right\rceil =6$, and the matrix in (4) becomes the following.

(5) $$\begin{array}{c} B^{\left(u\right)}=\left[\begin{array}{cccccccc} 0 & 0 & 0 & 0 & 1 & 1 & 1 & 1\\ 0 & 0 & 1 & 1 & 0 & 0 & 1 & 1\\ 0 & 1 & 0 & 1 & 0 & 1 & 0 & 1\\ 1 & 1 & 1 & 1 & 0 & 0 & 0 & 0\\ 1 & 1 & 0 & 0 & 1 & 1 & 0 & 0\\ 1 & 0 & 1 & 0 & 1 & 0 & 1 & 0 \end{array}\right]. \end{array}$$

Finally, matrices S and R are defined as in Section 3.1.2. Note that *D* is not set to be d+h here.

We are now ready to generate a filtering matrix and a scrutinizing matrix. The filtering matrix corresponding to matrix $M^{\left(u\right)}$ is as follows:

(6) $$F^{\left(u\right)}=\left[\begin{array}{c} M^{\left(u\right)}\left(1,:\right)\\ B^{\left(u\right)}\times \mathrm{diag}\left(M^{\left(u\right)}\left(1,:\right)\right)\\ \vdots \\ M^{\left(u\right)}\left(m_{u},:\right)\\ B^{\left(u\right)}\times \mathrm{diag}\left(M^{\left(u\right)}\left(m_{u},:\right)\right) \end{array}\right],$$

where diag(·) is a diagonal matrix generated by the input vector.

The vector observed after performing the tests given by the measurement matrix $F^{\left(u\right)}$ is described as follows:

(7) $$\begin{array}{c} y^{\left(u\right)}=F^{\left(u\right)}⊙x^{\left(u\right)}=\left[\begin{array}{c} M^{\left(u\right)}\left(1,:\right)⊙x^{\left(u\right)}\\ B^{\left(u\right)}⊙x_{1}^{\left(u\right)}\\ \vdots \\ M^{\left(u\right)}\left(m_{u},:\right)⊙x^{\left(u\right)}\\ B^{\left(u\right)}⊙x_{m_{u}}^{\left(u\right)} \end{array}\right]=\left[\begin{array}{c} y_{1}^{\left(u\right)}\\ y_{1}^{\left(u\right)}\\ \vdots \\ y_{m_{u}}^{\left(u\right)}\\ y_{m_{u}}^{\left(u\right)} \end{array}\right] \end{array}$$

where $x_{i}^{\left(u\right)}=\mathrm{diag}\left(M^{\left(u\right)}\left(i,:\right)\right)\times x^{\left(u\right)}$, $y_{i}=M^{\left(u\right)}\left(i,:\right)⊙x^{\left(u\right)}$, and $y_{i}^{\left(u\right)}=B^{\left(u\right)}⊙x_{i}^{\left(u\right)}$, for $i=1,2,\ldots ,m_{u}$. Entry $y_{i}$ indicates whether there exist only negatives and positives in that test. If the answer is yes, vector $y_{i}^{\left(u\right)}$ tells us whether there exists only one positive or more than one positive.

Let $\mathrm{expand}(M^{\left(u\right)}\left(i,:\right))$ be $M^{\left(u\right)}\left(i,:\right)$. Then, for any $j\in N^{\left(u\right)}$ and $M^{\left(u\right)}\left(i,j\right)=1$, every entry in $\mathrm{expand}(M^{\left(u\right)}\left(i,:\right))$ indexed from max{j−D+1,1} to min{j+D−1,n} is set to be 1. This vector is used to identify a block of 2D−1 consecutive items that contains at least one positive item. In particular, to identify positives only, the scrutinizing matrix corresponding to matrix $M^{\left(u\right)}$ isdefined as follows:

(8) $$S^{\left(u\right)}=\left[\begin{array}{c} S\times \mathrm{diag}(\mathrm{expand}\left(M^{\left(u\right)}\left(1,:\right)\right))\\ \vdots \\ S\times \mathrm{diag}(\mathrm{expand}\left(M^{\left(u\right)}\left(m_{u},:\right)\right)) \end{array}\right],$$

where S is defined in Section 3.1.2, and the outcome vector obtained by using this matrix is as follows:

(9) $$\begin{array}{c} s^{\left(u\right)}=S^{\left(u\right)}⊙x=\left[\begin{array}{c} S⊙(\mathrm{diag}\left(\mathrm{expand}\left(M^{\left(u\right)}\left(1,:\right)\right)\right)\times x)\\ \vdots \\ S⊙(\mathrm{diag}\left(\mathrm{expand}\left(M^{\left(u\right)}\left(m_{u},:\right)\right)\right)\times x), \end{array}\right]=\left[\begin{array}{c} s_{1}^{\left(u\right)}\\ \vdots \\ s_{m_{u}}^{\left(u\right)} \end{array}\right], \end{array}$$

where $s_{i}^{\left(u\right)}=S⊙\left(\mathrm{diag}\left(\mathrm{expand}\left(M^{\left(u\right)}\left(i,:\right)\right)\right)\times x\right)$, for $i=1,2,\ldots ,m_{u}$.

To identify both positives and inhibitors, an additional scrutinizing (n,kD−2,2)-disjunct matrix R is used. Let r be the outcome vector by using this matrix.

### 5.2. Decoding Procedure and Correctness

For each u∈{1,D}, we first scan each $y^{\left(u\right)}$ to locate some positive item in some block. The decoding procedure is as follows. First, find $1\leq i\leq m_{u}$ such that $y_{i}^{\left(u\right)}=1$ and $|y_{i}^{\left(u\right)}|=\left\lceil \log n_{u}\right\rceil$. Second, let α be the corresponding decimal number of the first half of $y_{i}^{\left(u\right)}$, where $y_{i}^{\left(u\right)}=1$ and $|y_{i}^{\left(u\right)}|=\left\lceil \log n_{u}\right\rceil$. Then, similarly to the arguments in Section 3.2, since any matrix composed of 2D−1 consecutive columns in S is a permutation of a (2D−1)×(2D−1) identity matrix, one can identify which item is positive based on the corresponding outcome vector $s_{i}^{\left(u\right)}$. Finally, all inhibitors in a block can be identified by using $r_{i}^{\left(u\right)}$.

Such *i* always exists in the first step. Indeed, as proved in Section 5.1 that for each special block τ, there exists an index $u_{\tau }$ such that some positive item in that block belongs to $N^{\left(u_{\tau }\right)}$. Since each set $N^{\left(u_{\tau }\right)}$ contains up to *k* positives and inhibitors and $M^{\left(u_{\tau }\right)}$ is a *k*-disjunct matrix, there must exist row *i* such that $M^{\left(u_{\tau }\right)}\left(i,:\right)$ contains only that positive. Therefore, $y_{i}^{\left(u_{\tau }\right)}=1$ and $|y_{i}^{\left(u_{\tau }\right)}|=\left\lceil \log n_{u_{\tau }}\right\rceil$. Conversely, if $y_{i}^{\left(u\right)}=1$, there must exist at least one positive item in $N^{\left(u\right)}$ in test $M^{\left(u\right)}\left(i,:\right)$. Moreover, since $B^{\left(u\right)}⊙\left(\mathrm{diag}\left(M^{\left(u\right)}\left(i,:\right)\right)\times x^{\left(u\right)}\right)$ and every column in $B^{\left(u\right)}$ has weight of $\left\lceil \log n_{u}\right\rceil$, there must exist only one positive item in $N^{\left(u\right)}$ in that test. Otherwise, $|y_{i}^{\left(u\right)}|>\left\lceil \log n_{u}\right\rceil$.

In the second step, the indices of positives and inhibitors then ranged from max{1,α−D+1} to min{α+D−1,n}. Because of the construction of vector $\mathrm{expand}(M^{\left(u\right)}\left(1,:\right))$, every item indexed from max{1,α−D+1} to min{α+D−1,n} presents in the characteristic vector $\mathrm{diag}(\mathrm{expand}\left(M^{\left(u\right)}\left(1,:\right)\right))\times x$. Therefore, $s_{i}^{\left(u\right)}$ is the union of up to *D* columns in S, which out of them corresponds to all positives and inhibitors in a specific block. The positives are thus identified. The decoding complexity of $s_{i}^{\left(u\right)}$ is therefore O(D).

In the last step, since R is an (n,kD−2,2)-disjunct matrix, for any block of positives and inhibitors, there exists a row such that it contains only a positive and an inhibitor. That inhibitor is thus identified. This procedure takes $O\left(k\times r\left(2D-1\right)\right)=O\left(k^{4}D^{4}\log\left(n/(kD)\right)\right)$.

### 5.3. Decoding Complexity and Number of Tests

There are *D*$F^{\left(u\right)}$ deterministic and strongly explicit matrices in the filtering procedure. Since each has $m_{u}\left(1+b\right)=O\left(k^{2}\log n_{u}\times \log n_{u}\right)=O\left(k^{2}\log^{2}\left(n/D\right)\right)$ tests, the total number of tests in the filtering procedure is $O(Dk^{2}\log^{2}\left(n/D\right))$. The decoding complexity by using these tests is also $O(Dk^{2}\log^{2}\left(n/D\right))$.

There are also *D*$S^{\left(u\right)}$ matrices and *D*$R^{\left(u\right)}$ matrices. The total number of tests for *D*$S^{\left(u\right)}$ matrices and *D*$R^{\left(u\right)}$ matrices include $m_{u}D\left(2D-1\right)=O\left(m_{u}D^{2}\right)$ and $m_{u}Dr=O\left(m_{u}rD\right)$, respectively. Therefore, the number of tests for identifying positives only (both positives and inhibitors) is $O\left(Dm_{u}\left(1+b+s\right)\right)=O\left(Dk^{2}\log\left(n/D\right)\left(D+\log\left(n/D\right)\right)\right)$ (respectively, $O\left(Dm_{u}\left(1+b+s\right)+r\right)=O\left(k^{2}D^{2}\log\left(n/D\right)\left(\log\left(n/D\right)+kD\right)\right)$).

For each *u*, the running time to decode all $s_{i}^{\left(u\right)}$s is $O\left(m_{u}s\right)=O\left(Dk^{2}\log\left(n/D\right)\right)$. Since *u* ranges from 1 to *D*, the running time to find all positives is $O\left(Dk^{2}\log^{2}\left(n/D\right)\right)+D\times O\left(m_{u}s\right)=O\left(Dk^{2}\log\left(n/D\right)\left(D+\log\left(n/D\right)\right)\right)$. On the other hand, the running time to find all positives and inhibitors is as follows.

$$O\left(Dk^{2}\log\frac{n}{kD}\left(D+\log\frac{n}{D}\right)\right)+O\left(k^{4}D^{4}\log\frac{n}{kD}\right)=O\left(Dk^{2}\log\frac{n}{kD}(D+\log\frac{n}{D})+k^{4}D^{4}\log\frac{n}{kD}\right).$$

## 6. Discussion

### 6.1. Comparison

We compare our proposed schemes with existing schemes, namely Ganesan et al. [32], Chang et al. [33], and Bui et al. [20] in Table 1. There are eight criteria to consider here. The first four criteria are about the structure of the population of *n* items. They are the number of blocks, the number of items in a block, the number of positives (in a block if applicable), and the number of inhibitors (in a block if applicable). The fifth criterion is the decoding type. The sixth is the construction type, which describes how measurement matrices can be achieved. The seventh and the last are the number of tests and the decoding time.

Consider the decoding type as “positives only.” The construction type in our proposed schemes for the first and the second model, i.e., the number of blocks is one, is deterministic and strongly explicit. They are better than the schemes proposed by Chang et al. and Ganesan et al., whose schemes are random and explicit. The numbers of tests in our proposed schemes are almost less than a factor of d+h compared to the ones in Chang et al.’s and Bui et al.’s schemes and less than the one in Ganesan et al.’s scheme. More importantly, our decoding times scale to the number of tests while the ones in Chang et al.’s and Ganesan et al.’s schemes scale to the number of tests and the number of items. For the third model, the same arguments are applied by replacing *d* by dk and *h* by hk.

We now consider the decoding type as “positives and inhibitors.” For the first and second models, the number of tests in our proposed schemes is relatively the same as the one in Chang et al.’s scheme, smaller than the one in Bui et al.’s scheme, and less than the one in Ganesan et al.’s scheme. Meanwhile, the decoding times are smaller than the ones in the three existing schemes. For the third model, the number of tests in our proposed schemes is relatively similar to the one in Chang et al.’s scheme, smaller than the one in Bui et al.’s scheme, and larger than the one in Ganesan et al.’s scheme. However, our decoding time is smaller than the ones in the three existing works.

### 6.2. Potential Applications

Bruno et al. [34] addressed a group testing-based solution in genetic mapping and sequencing. In this application, the authors consider linear DNA, which consists of consecutive segments of the DNA. Each segment is placed in a pool, called clones, in an order consistently to the order of their appearance in the linear DNA. A collection of such clones is called a linear DNA library. From this point, we can ask where segments (clones) of interest are in the linear DNA library [35]. The segments of interest here can be considered as positives and other segments can be considered as negatives. A pool that contains at least one segment of interest returns a positive outcome when performing testing and returns a negative outcome otherwise.

We extend the application above to a potential application as follows. Given a linear DNA library, we would like to find segments of DNA that express a certain biological property and segments of DNA that inhibit the segments expressing a certain biological property. The first and second types of segments of interest are considered as positives and inhibitors, respectively, while the remaining segments are considered as negatives. Because of the nature of DNA, an inhibitor is usually close to positives. Therefore, the blocks of positives and inhibitors model can be used to identify both positives and inhibitors.

## 7. Conclusions

In this paper, we presented efficient encoding and decoding procedures to identify positives and/or inhibitors in a single block of (consecutive) positives and inhibitors or in blocks of positives and inhibitors. The number of tests and the decoding times in our proposed schemes is usually smaller than the ones in existing works. An extension of this work to other settings in group testing such as threshold group testing or complex group testing is still an open problem.

## Figures and Tables

**Table 1:** Comparison with previous work. “Det.” and ”Rnd.” stand for “Deterministic” and “Random.” We set $\lambda =\frac{(d+h)\ln n}{W((d+h)\log n)}$, $\alpha =\max\left\{\frac{\lambda }{{\left(d+h\right)}^{2}},1\right\}$, and $\beta =O(Dk^{2}\left(D+\log\frac{n}{D}\right)\log\frac{n}{D}+k^{4}D^{4}\log\frac{n}{kD})$, where $W\left(x\right)e^{W(x)}=x$ and W(x)∼Θ(logx−loglogx).

No. of Blocks	No. of Items in a Block	No. of Positives (in a Block)	No. of Inhibitors (in a Block)	Decoding Type	Scheme	Construction Type	No. of Tests *t*	Decoding Complexity
Not applicable		*d*	*h*	Positives only	Ganesan et al. [32]	Rnd., Explicit	O((d+h)logn)	O(tn)
		≤d	≤h		Chang et al. [33]		$O({\left(d+h\right)}^{2}\log n)$	O(tn)
					Bui et al. [20]	Det., Strongly explicit	$O(\lambda^{2}\log n)$	$O\left(\frac{\lambda^{5}}{{\left(d+h\right)}^{2}}\right)$
1	d+h	*d*	*h*		**Theorem 1**		$O\left(h\log\frac{hn}{d+h}+d\right)$	O(t)
	*D*	≤d	≤h		**Theorem 1**		O(Dlogn)	O(t)
*k*	≤D	≤d	≤h		**Theorem 2**	Rnd., Explicit	$O(Dk^{2}\left(D+\log\frac{n}{D}\right)\log\frac{n}{D})$	O(t)
Not applicable		*d*	*d*	Positives and inhibitors	Ganesan et al. [32]	Rnd., Explicit	$O(\left(d+h^{2}\right)\log n)$	O(tn)
		≤d	≤h		Chang et al. [33]	Rnd., Explicit	$O({\left(d+h\right)}^{3}\log n)$	O(tn)
					Bui et al. [20]	Det., Strongly explicit	$O(\lambda^{3}\log n)$	$O(d\lambda^{6}\alpha )$
1	d+h	*d*	*h*		**Theorem 1**	Rnd., Explicit	$O\left({\left(d+h\right)}^{3}\log\frac{n}{d+h}\right)$	$O\left({\left(d+h\right)}^{4}\log\frac{n}{d+h}\right)$
	*D*	≤d	≤h		**Theorem 1**		$O\left(D\log n+D^{3}\log\frac{n}{D}\right)$	$O\left(D\log n+D^{4}\log\frac{n}{D}\right)$
*k*	≤D	≤d	≤h		**Theorem 2**	Rnd., Explicit	$O(D^{2}k^{2}\left(D+\log\frac{n}{D}\right)\log\frac{n}{D})$	β

## Data Availability

Not applicable.
